# Multi-Modal Explicit Sparse Attention Networks for Visual Question Answering

**DOI:** 10.3390/s20236758

**Published:** 2020-11-26

**Authors:** Zihan Guo, Dezhi Han

**Affiliations:** College of Information Engineering, Shanghai Maritime University, Shanghai 201306, China; guozihan0006@stu.shmtu.edu.cn

**Keywords:** attention mechanism, computer vision, natural language processing, sparse attention, visual question answering

## Abstract

Visual question answering (VQA) is a multi-modal task involving natural language processing (NLP) and computer vision (CV), which requires models to understand of both visual information and textual information simultaneously to predict the correct answer for the input visual image and textual question, and has been widely used in smart and intelligent transport systems, smart city, and other fields. Today, advanced VQA approaches model dense interactions between image regions and question words by designing co-attention mechanisms to achieve better accuracy. However, modeling interactions between each image region and each question word will force the model to calculate irrelevant information, thus causing the model’s attention to be distracted. In this paper, to solve this problem, we propose a novel model called Multi-modal Explicit Sparse Attention Networks (MESAN), which concentrates the model’s attention by explicitly selecting the parts of the input features that are the most relevant to answering the input question. We consider that this method based on top-k selection can reduce the interference caused by irrelevant information and ultimately help the model to achieve better performance. The experimental results on the benchmark dataset VQA v2 demonstrate the effectiveness of our model. Our best single model delivers 70.71% and 71.08% overall accuracy on the test-dev and test-std sets, respectively. In addition, we also demonstrate that our model can obtain better attended features than other advanced models through attention visualization. Our work proves that the models with sparse attention mechanisms can also achieve competitive results on VQA datasets. We hope that it can promote the development of VQA models and the application of artificial intelligence (AI) technology related to VQA in various aspects.

## 1. Introduction

Recently, multi-modal learning tasks such as image captioning [1,2], image-text matching [3,4,5], and visual question answering (VQA) [6], which involve natural language processing and computer vision, have attracted considerable attention of researchers in these two fields. Compared with other multi-modal learning tasks, VQA is more difficult, since it requires the model to understand visual information, textual information, and the relationships between them simultaneously, and may also require complex reasoning and commonsense knowledge to correctly answer the questions. Therefore, VQA is regarded as a benchmark for general artificial intelligence (AI). The vigorous development of AI technology has brought great changes in sensors related fields. The perfect combination of sensors and deep learning enables the machine to have senses such as vision, hearing and smell, which makes it possible for high-value sensor data analysis and low-cost, real-time intelligent sensor systems.

A simple instance of a VQA dataset contains a visual image and a textual question related to the content of the image, which requires the model to predict the correct answer for the input image and question. In practice, VQA also has many applications, such as helping researchers to carry out image retrieval [7], providing aided-navigation for blind individuals [8], helping humans to implement smart and intelligent transport systems and smart city, being used in the field of sensor networks, etc. For example, when used to build smart and intelligent transport systems, VQA can help researchers to process and analyze the massive information collected by sensors and other information collection equipment. Specifically, the VQA model takes the image information collected by monitors and the corresponding questions as the main input, and takes other information such as the speed of vehicles per hour and traffic information collected by sensors and other collectors as the secondary input to predict the answers that will help traffic management, providing traffic information services, etc. With the development of various components of VQA models such as feature extractors, attention mechanisms, and feature fusion methods, the performance of VQA models and their performance in various intelligent tasks are also improving.

Attention mechanism is an advanced technology in deep neural networks, which is widely applied to machine translation [9], speech recognition [10], image captioning, and other fields. Attention mechanism helps models to assign different weights to the features of different importance to make the learning of neural network more flexible. The first method to introduce visual attention into VQA was proposed by [11]. After that, attention mechanism has become an inherent part of VQA models [12,13,14]. Visual attention assigns different weights to different regions of the image to make the model focus on the image regions that help to answer the question correctly. Similarly, learning textual attention that can enable the model to focus on the question key words is also very important to VQA models that need to understand textual information. Recently, co-attention that combines visual attention and textual attention has proved that VQA models which can focus on important image regions and question key words simultaneously have better performance [15,16,17,18,19,20], and it has become the main attention mechanism used in VQA models.

Most of the early co-attention models learned the coarse interactions between image regions and question words but ignored the dense interactions between them. Two dense co-attention models DCN (dense co-attention network) [21] and BAN (bilinear attention network) [5] proposed later improved the performance of VQA model by modeling dense interactions between each image region and each question word. However, due to the lack of modeling self-attention within each modality (i.e., region-to-region interactions for images and word-to-word interactions for questions), the deep stacked versions of these co-attention models showed little improvement over their corresponding shallow models. Inspired by the transformer model [22], some recently proposed models such as MCAN (modular co-attention network) [23] and MEDAN (multi-modal encoder-decoder attention networks) [24] can model the dense intra-model interactions (region-to-region or word-to-word) and inter-model interactions (word-to-region) simultaneously, thus achieving the best level of accuracy. The experimental results showed that these models can achieve deep reasoning by deep stacking their basic modular co-attention layers.

However, modular co-attention models like MCAN and MEDAN, which model interactions between each image region and each question word, will force the model to calculate irrelevant information, thus causing the model’s attention to be distracted. If irrelevant information imposes negative impacts on reading comprehension, retrieval problems may occur. Such distraction will hinder the understanding process, thus VQA models need effective attention mechanisms. Inspired by the Explicit Sparse Transformer [25], we propose a novel model called Multi-modal Explicit Sparse Attention Networks (MESAN) that employs explicit selection based on top-k selection to focus only on the specified number of question words that are the most relevant to answering the question. We consider that this method can reduce the interference caused by irrelevant information and ultimately help the model to achieve better performance. Extensive experimental results on the benchmark dataset VQA v2 [26] prove the effectiveness of our models and show that our models can achieve state-of-the-art VQA performance. Our best single model delivers 70.71% and 71.08% overall accuracy on the test-dev and test-std sets, respectively. We also explore the effectiveness of our models through ablation experiments and demonstrate that our models can obtain better attended features than other advanced VQA models through attention visualization.

The rest of this paper is organized as follows: Section 2 introduces researches related to VQA and the framework and details of MESAN. The dataset, experimental deployment, and experimental results are described in Section 3. In Section 4, we detail the comparison results and attention visualization results. Section 5 specifies the conclusion and future works.

## 2. Materials and Methods

### 2.1. Related Work

We briefly introduce the research on VQA, especially those that introduce co-attention mechanisms.

#### 2.1.1. Visual Question Answering

VQA is a widely studied vision-and-language problem that requires models to understand of both image content and natural language question as well as their relationship simultaneously, and may also need complex reasoning and commonsense knowledge. The first step in most VQA models [12,27,28,29,30,31,32,33] is to use feature extractors to extract features from the input image and question. Then, the model uses a fusion method to fuse the extracted image features and question features. Finally, a classifier takes the joint embedding as input to predict the correct answer.

However, the method described above is based on global features. The use of global features of the input image and question will make it difficult for the model to focus on the image regions and question key words that are the most relevant to answering the question and may introduce noise. Therefore, some researches have introduced visual attention mechanisms into VQA based on the assumption that humans can quickly understand the visual scene by focusing on local regions of the image instead of processing the whole scene at once. Yang et al. [34] proposed a stacked attention network that used the semantic representation of the input question to search for the local image regions related to answering the question to carry out multi-step reasoning on the input image to predict the correct answer. Anderson et al. [14] designed a bottom-up mechanism to detect image regions and a top-down attention to assign different weights to the feature vectors related to the regions. Schwartz et al. [35] proposed an attention mechanism that learned high-order correlations between various data modalities for VQA, which can effectively guide the model to focus on the elements in the different data modalities that were required to solve the multi-modal task.

#### 2.1.2. Co-Attention Models

In addition to visual attention, textual attention that can guide models to focus on question key words is also very important, because VQA requires the model to understand of both visual information and textual information simultaneously. Therefore, co-attention mechanisms that combine visual attention and textual attention have become an import component of advanced VQA models. The Dual Attention Network (DAN) proposed in [4] gathered essential information by attending to specific regions in images and key words in questions through multiple steps. Lu et al. [15] designed the Hierarchical Co-attention Model (HieCoAtt) to alternatively learn visual attention and textual attention. However, the aforementioned early co-attention models only learned the coarse interactions between image regions and question words but ignored the dense interactions between them. Two dense co-attention models DCN [21] and BAN [5] proposed later improved this defect by modeling dense interactions between each image region and each question word. Although these dense co-attention models can be cascaded in depth, their deep stacked versions have few performance advantages. Recently proposed models such as MCAN and MEDAN achieve deep reasoning by modeling the dense intra-model interactions (region-to-region or word-to-word) and inter-model interactions (word-to-region), thus improving the accuracy of VQA models to a new level.

### 2.2. Multi-Modal Explicit Sparse Attention Networks

**Figure 1:**
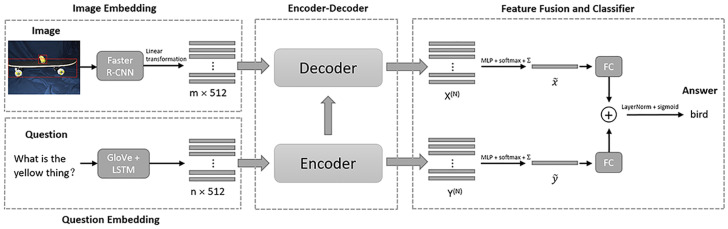
The overall architecture of Multi-modal Explicit Sparse Attention Networks (MESAN).

In this section, we first introduce how we extract the image features and question features. Then we describe the explicit sparse attention and encoder-decoder strategy used in our model. Finally, we employ a fusion mechanism to obtain the joint embedding of the attended image features and attended question features, and feed it into a classifier to predict the most likely answer. The overall architecture of MESAN is given in Figure 1.

#### 2.2.1. Image and Question Embeddings

Following [14], we employ the bottom-up and top-down visual attention mechanism to extract image features. We use the Faster R-CNN model [35] to propose a set of image regions that is represented by pooled convolutional feature vectors. The extracted image features are represented as $X\in R^{m\times 2048}$, where m∈[10,100] is the number of detected objects. In practice, we use a linear transformation of *X* to make its dimension consistent with the question features. Therefore, the dimension of the image features we used becomes m×512 as shown in Figure 1.

For the input question, we first tokenize it into words and trim it to a maximum of 14 words the same as [5,23]. Then we use the 300-D GloVe word embeddings [36] pre-trained on a large-scale corpus to transform the question words into a sequence of word embeddings of size n×300, where n∈[1,14] is the number of question words. Finally, we use a single layer 512-dimensional long short-term memory (LSTM) to encode the word embeddings into the question features $Y\in R^{n\times 512}$.

In practice, we use zero-padding to fill *X* and *Y* to their maximum (i.e., *m* = 100 and *n* = 14) to handle the variable number of image objects and question words. During training, the padding logits are masked with −∞ to avoid the underflow problem.

#### 2.2.2. Explicit Sparse Attention

Our model is composed of an encoder and a decoder. The core of the encoder is stacked SA (self-attention) units that can learn self-attention for question words, and the core of the decoder is stacked SGA (self&guided-attention) units that can learn self-attention and question-guided attention for input images. The SA and SGA units are inspired by the scaled dot-product attention proposed in [22], and the difference is that we implement an explicit selection based on top-k selection to obtain more concentrated attention.

1. Sparse Scaled Dot-Product Attention

Given a query and a set of key-value pairs, an attention mechanism can map them to an output. The query, keys, values, and output are all vectors and the output is a weighted sum of the values, where weight is computed by a compatibility function of the query with the corresponding key. The inputs to sparse scaled dot-product attention include the query $Q[l_{Q},d]$, key $K[l_{K},d]$, and value $V[l_{V},d]$ and they are the linear transformation of the image features and question features. We first compute the dot products of *Q* and *K*, and divide by $\sqrt{d}$ to obtain the attention scores *P*:

(1) $$P=\frac{QK^{T}}{\sqrt{d}}$$

We evaluate the scores *P* based on the assumption that scores with larger values demonstrate higher relevance. Then we implement sparse attention masking function M(·) on *P* to explicitly select the top-k contributive elements. The function M(·) selects the *k* largest elements of each row in *P* and records their position in a position matrix. Specifically, it first selects the *k*-th largest value of row *i* and mark it as $a_{i}$. If the value of the *j*-th element is larger than $a_{i}$, the position (i,j) is recorded. The sparse attention masking function M(·) is as follows:

(2) $$M{\left(P,k\right)}_{ij}=\left\{\begin{array}{c} P_{ij}\;if\;P_{ij}\geq a_{i}\\ -\infty \;if\;P_{ij}<a_{i} \end{array}\right.$$

Unlike Dropout [37], we only explicitly select the high attention scores through top-*k* selection. The function M(·) assigns negative infinity with the scores that are smaller than the top *k* largest scores and thus, the corresponding probabilities approximate 0. *k* is a hyperparameter and is usually a small number. Then we apply a softmax function to get the weights on the value:

(3) W=softmax(M(P,k))

Finally, the attended features *F* is given by:

(4) F=WV

**Figure 2:**
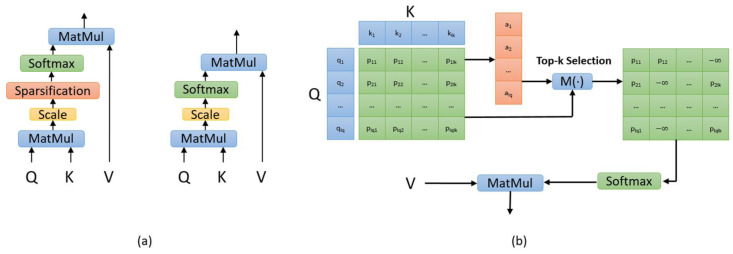
(**a**) The difference between sparse scaled dot-product attention (left) and ordinary dot-product attention (right). (**b**) The core calculation steps of sparse scaled dot-product attention. Through explicit selection based on top-k selection and softmax function, only the most contributive elements are assigned with probabilities.

By employing sparse scaled dot-product attention, we can not only obtain more concentrated attention, but also eliminate the negative impacts imposed by irrelevant segments. Figure 2a shows the difference between sparse scaled dot-product attention and ordinary scaled dot-product attention, and Figure 2b describes the core calculation steps of sparse scaled dot-product attention.

2. Multi-Head Sparse Attention

Multi-head attention [22] allows the model to jointly attend to information from different representation subspaces at different positions to improve the representation capacity of the attended features. Using the same idea, our multi-head sparse attention has *h* parallel attention heads and each head corresponds to an independent sparse scaled dot-product attention function. The multi-head sparse attention is formulated as:

(5) $$Multihead\left(Q,K,V\right)=Concat\left(head_{1},\ldots ,head_{h}\right)W^{O}$$

(6) $$where\;head_{i}=softmax\left(M\left(\frac{QW_{i}^{Q}{\left(KW_{i}^{K}\right)}^{T}}{\sqrt{d}},k\right)\right)\left(VW_{i}^{V}\right)$$

where $W_{i}^{Q},W_{i}^{K},W_{i}^{V},W^{O}$ are the projection matrices.

#### 2.2.3. Encoder and Decoder

It has been proved in [23] that the performance of the encoder-decoder model is better than that of the stacking model, thus we also use the encoder-decoder structure in our model.

- Encoder: We use encoder to implement self-attention to learn fine-grained question features. The encoder consists of *N* stacked identical SA (self-attenion) units and each SA unit has two sub-layers. The first sub-layer is a multi-head sparse attention layer and the second is a pointwise fully connected feed-forward layer. The first SA unit takes question features $Y\left[y_{1};\ldots ;y_{n}\right]\in R^{n\times 512}$ as input, and its multi-head sparse attention layer learns the correlation between each word pair $<y_{i},y_{j}>$. The feed-forward layer of the first SA unit further transforms the output of its previous sub-layer through two fully connected layers with ReLu [38] and Dropout and outputs the attended question features. Every other SA unit takes the output of its previous SA unit as input and we mark the output of the encoder as $Y^{\left(N\right)}\left[y_{1}^{\left(N\right)};\ldots ;y_{n}^{\left(N\right)}\right]\in R^{n\times 512}$.
- Decoder: The decoder consists of *N* stacked identical SGA (self&guided-attention) units, each of which has three sub-layers and outputs the attended image features. The first and the second sub-layers are both a multi-head sparse attention layer with sparse scaled dot-product attention as the core. The first sub-layer of the first SGA unit takes image features $X\left[x_{1};\ldots ;x_{m}\right]\in R^{m\times 2048}$ as input and every other SGA unit takes the output attended image features of its previous SGA unit as input to its first sub-layer. In practice, we use a linear transformation of *X* to make its dimension consistent with the question features. The second sub-layer takes the attended image features obtained from its previous sub-layer and the output of the encoder, i.e., the attended question features $Y^{\left(N\right)}\left[y_{1}^{\left(N\right)};\ldots ;y_{n}^{\left(N\right)}\right]\in R^{n\times 512}$ as input to learn question-guided attention for the input image features. The last sub-layer is a feed-forward layer, which is the same as that in the encoder and it also takes the output of its previous sub-layer as input. We mark the output of the decoder as $X^{\left(N\right)}\left[x_{1}^{\left(N\right)};\ldots ;x_{m}^{\left(N\right)}\right]\in R^{m\times 512}$.

**Figure 3:**
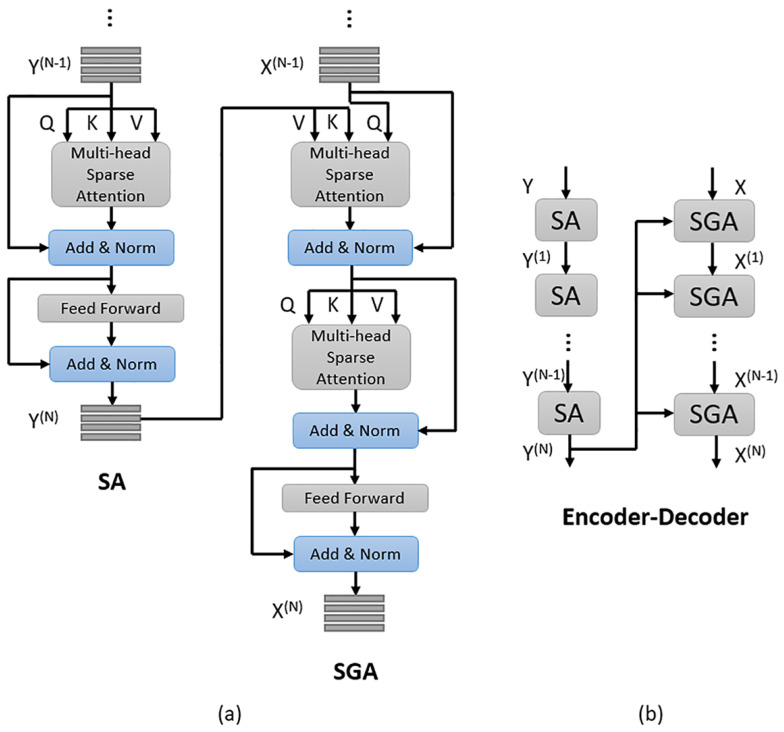
(**a**) The details of SA (slef-attention) and SGA (self&guided-attention) units. (**b**) The structure of the encoder-decoder model.

To facilitate optimization, we also apply residual connection [39] followed by layer normalization [38] to the outputs of each sub-layer of SA and SGA. Figure 3 shows the details of SA and SGA units, and the structure of the encoder-decoder model.

#### 2.2.4. Feature Fusion and Classifier

After the encoder-decoder learning stage, we obtain the attended question features $Y^{\left(N\right)}\left[y_{1}^{\left(N\right)};\ldots ;y_{n}^{\left(N\right)}\right]\in R^{n\times 512}$ and attended image features $X^{\left(N\right)}\left[x_{1}^{\left(N\right)};\ldots ;x_{m}^{\left(N\right)}\right]\in R^{m\times 512}$. We design a two-layer MLP (FC(512)-ReLu-Dropout(0.1)-FC(1)) for $Y^{\left(N\right)}$ and $X^{\left(N\right)}$ to compute their final attended features as follows:

(7) $$\alpha =softmax(MLP\left(X^{\left(N\right)}\right))$$

(8) $$\tilde{x}=\sum_{i=1}^{m}\alpha_{i}x_{i}^{\left(N\right)}$$

where $\alpha =\left[\alpha_{1},\ldots ,\alpha_{m}\right]\in R^{m}$ are the attention weights and $\tilde{x}\in R^{512}$ is the attended image feature. We use the same method to obtain the attended question feature $\tilde{y}\in R^{512}$. The feature fusion mechanism is as follows:

(9) $$Z=LayerNorm(W_{x}^{T}\tilde{x}+W_{y}^{T}\tilde{y})$$

where *Z* is the fused feature, LayerNorm [38] is used to optimize training and $W_{x},W_{y}\in R^{512\times 1024}$ are two linear projection matrices. Finally, *Z* is projected into a vector $s\in R^{A}$ followed by a sigmoid function [40], where *A* is the number of the most frequent answers in training set. The sigmoid function is used as a classifier to obtain the final result.

## 3. Results

We perform extensive experiments and ablation studies on the largest VQA dataset VQA v2 [26] to explore the effectiveness of our model and experimentally answer how to choose the appropriate *k*. In this section, we will introduce the benchmark dataset VQA v2 and describe the details of our experiments.

### 3.1. The Dataset

To solve the language priors in VQA, Goyal et al. [26] balance the VQA dataset [6] by collecting complementary images for each question such that every question in VQA v2 is associated with a pair of similar images that results in two different answers to the question. The complementary images were collected from Amazon Mechanical Turk (AMT) and a second round of data annotation was conducted to collect answers on these new images. Thus, VQA v2 is more balanced and is approximately twice the size of the original VQA dataset. VQA v2 is divided into train set, validation set, and test set, and the test set is further split into test-dev and test-std to evaluate VQA models online. The answers are divided into three types: Yes/No, Number, and Other.

### 3.2. Experimental Setup

The dimensions of the image features, question features, and fused multi-modal features are 2048, 512, and 1024, respectively. The dimension of the multi-head sparse attention $d_{m}$ is 512 and the number of head *h* is set to 8. To limit the size of the multi-head sparse attention module, the dimension of the output features of each head is set to $d_{m}/h$ = 64. Following the strategy in [41], the number of the most frequent answers in the training set is 3129, i.e., *A* = 3129. Considering the training time and the number of parameters, we set the number of the stacked layers of SA and SGA units to 6, i.e., *N* = 6, according to the suggestion in [23,24].

All the models in this paper are trained with the Adam optimizer [42] with $\beta_{1}=0.9,\beta_{2}=0.98$, and the batch size is set to 64. The warm-up learning rate is set to min$\left(2.5te^{-5},1e^{-4}\right)$, where *t* is the current epoch number starting from 1. The dropout in all fully connected layers is set to 0.1. The learning rate decays by factor 0.2 every 2 epochs after 10 epochs. All the models are trained up to 13 epochs. In addition, we use a subset from Visual Genome [43] as a dataset for auxiliary training. The experimental results of other advanced VQA models show that this can improve the overall performance of the models.

### 3.3. Experimental Results and Ablation Studies

**Figure 4:**
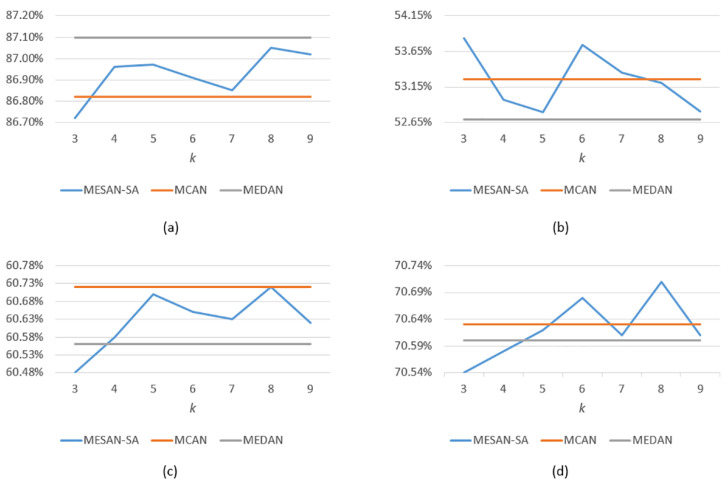
(**a**) The Yes/No accuracy of MCAN, MEDAN, and MESAN-SA with different *k*. (**b**) The Number accuracy of MCAN, MEDAN, and MESAN-SA with different *k*. (**c**) The Other accuracy of MCAN, MEDAN, and MESAN-SA with different *k*. (**d**) The Overall accuracy of MCAN, MEDAN, and MESAN-SA with different *k*. All the reported results are evaluated on VQA v2 test-dev set.

**Figure 5:**
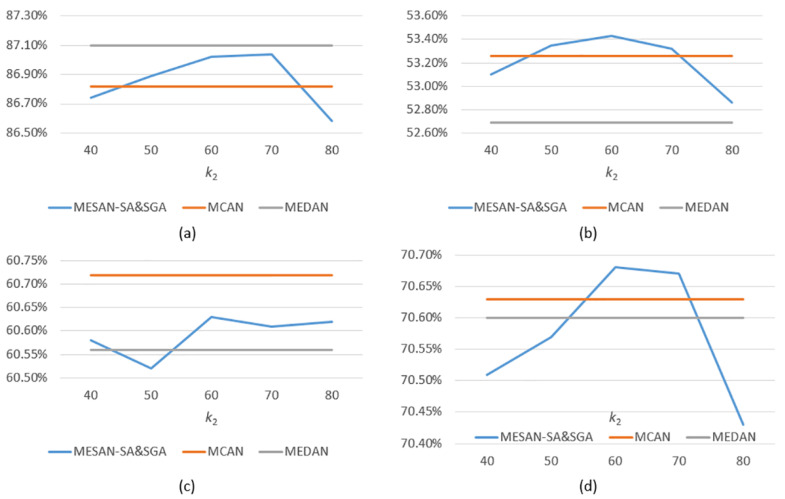
(**a**) The Yes/No accuracy of MCAN, MEDAN, and MESAN-SA&SGA with different $k_{2}$. (**b**) The Number accuracy of MCAN, MEDAN, and MESAN-SA&SGA with different $k_{2}$. (**c**) The Other accuracy of MCAN, MEDAN, and MESAN-SA&SGA with different $k_{2}$. (**d**) The Overall accuracy of MCAN, MEDAN, and MESAN-SA&SGA with different $k_{2}$. All the reported results are evaluated on VQA v2 test-dev set.

We conduct extensive experiments and ablation studies on VQA v2 to explore the performance of our models. In order to limit the size of the models and save computing time, we set the appropriate stacked layers *N* of attention units and the number of head *h* of multi-head sparse attention, according to the experience of MCAN and MEDAN. Therefore, we only need to explore the effectiveness of sparse attention networks with different variants and choose the appropriate *k* to make the models achieve the best performance. The results are shown in Table 1, Figure 4, Table 2, and Figure 5. And the best results in the tables are bold.

- MESAN-SA: MESAN-SA means that only the SA units for learning question self-attention in encoder adopt explicit sparse attention, while the SGA units in decoder adopt the ordinary scaled dot-product attention. The length of the input question words is 14, thus we need to select k∈ [1, 14] most relevant question key words for subsequent experiments. During ablation studies, we evaluate the performance of k∈ {3, 4, 5, 6, 7, 8, 9}. From Table 1 and Figure 4d, we can see that the accuracy of the model roughly increases first and then decreases with the increase of *k*. When *k* = 8, the model achieves the highest accuracy, 70.71%.
- MESAN-SA&SGA: MESAN-SA&SGA means that both the SA units in encoder and the first sub-layer for learning image self-attention in SGA in decoder adopt explicit sparse attention, while the second sub-layer for learning question-guided attention in SGA adopts the ordinary scaled dot-product attention. Considering that the input features of the second sub-layer in SGA are selected by top-k selection, we no longer use sparse attention in it. The number of regions of the input image is 100, thus we need to select $k_{2}\in$ [1, 100] most relevant image regions for subsequent experiments. During ablation studies, we set the parameter $k_{1}$ of top-k selection used in encoder to 6 and evaluate the performance of different $k_{2}\in$ {40, 50, 60, 70, 80} of top-k selection used in decoder. From Table 2 and Figure 5d, we can see that the performance of the model rises first and then falls as $k_{2}$ increases. When $k_{2}$ = 60, the model achieves the highest accuracy, 70.68%.

The difference between the two models is that the former only uses sparse attention in encoder, while the later also uses sparse attention in decoder. From Table 1 and Table 2, we can see that the performance of MESAN-SA, which uses original scaled dot-product attention to learn visual self-attention, is better than that of using sparse attention to learn visual self-attention. We think that this is because image features are more complex and have more noise than textual features. Therefore, using sparse attention to learn visual self-attention will have a negative impact on the performance of the model.

## 4. Discussion

We also compare MESAN with other advanced VQA models through experimental results and attention visualization.

### 4.1. Comparison with Advanced VQA Models

We compare the performance of our models with previous published competing methods. Table 3 shows the experimental results on the VQA v2 dataset with test-dev and test-std sets. Using Faster R-CNN based bottom-up attention to extract image region features is one of the most common and advanced method used by VQA models. Therefore, for fair comparison, the models in Table 3 use the image features extracted by bottom-up attention and are all a single network. Among them, bottom-up [41] is the most basic VQA model that uses bottom-up attention. MFH (generalized multi-modal factorized high-order pooling approach) [16] was developed by cascading multiple Multi-modal Factorized Bilinear Pooling (MFB) blocks to achieve more effective fusion of the visual features and textual features, which won the runner-up in VQA Challenge 2017 and also used co-attention mechanism. BAN [5] achieved the first place in the leaderboard of both VQA Challenge 2017 and 2018 at the time of submission by learning and using bilinear attention distributions. Moreover, it further used the counting module [44], which exploited auxiliary spatial information to further improve the accuracy of the model on counting problems. MCAN and MEDAN are the most advanced co-attention models, and our models are also based on co-attention mechanism. As can be seen from Table 3, our best model MESAN-SA(*k* = 8) achieves the highest overall accuracy on both test-dev and test-std sets. On Yes/No and Number questions, although our models are not the best, they also reach the similar accuracy levels. We can see from Table 1 and Table 2 that our MESAN-SA(*k* = 6) and MESAN-SA&SGA($k_{1}=6,k_{2}=70$) also achieve better results than MCAN and other models in Table 3. We have done five-fold experiments on MESAN-SA(*k* = 8), and the lowest accuracy on test-dev is 70.66%, which is also higher than that of MCAN and MEDAN(Adam). In addition, we also replace the scaled dot-product attention in MEDAN for learning question self-attention with sparse attention and have carried on experiment verification. The results are shown in Table 4. We can see that when *k* = 5, the accuracy of the model is improved on all kinds of questions except Yes/No questions, and the accuracy on Yes/No questions is only reduced by 0.15%. We have done five-fold experiments on MEDAN-Sparse(*k* = 5), and the lowest accuracy on test-dev is 70.60%. This is the same as the result of MEDAN(Adam), but the results of the other four time experiments on this model are higher than this value. This is a good proof of the robustness of our method that using explicit sparse attention on VQA models. All the above experimental results, including ablation experiments, prove the effectiveness of our models.

### 4.2. Attention Visualization

**Figure 6:**
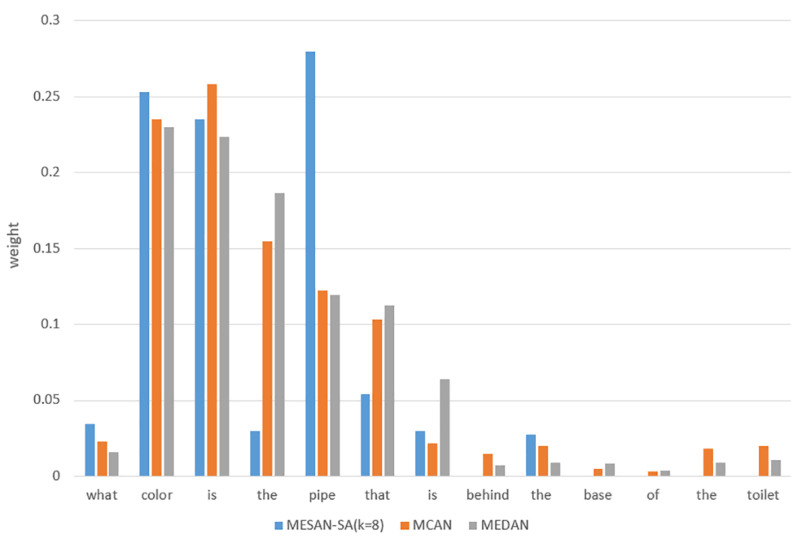
The visualization results of the attention of MESAN-SA(*k* = 8), MCAN, and MEDAN for a specific question. The ordinate is the weight to measure the importance of a word.

Figure 6 shows the visualization results of the attention of MESAN-SA(*k* = 8), MCAN and MEDAN for a specific question. We can see that MCAN and MEDAN assign a portion of weight to words that are not related to answering the question, while MESAN focuses on the most relevant *k* words by explicit selection. For the key words “color” and “pipe” of this question, MESAN also gives higher weights than MCAN and MEDAN, which can help the model to focus on the specified number of question key words to better predict the answer. We think that this method can reduce the noise caused by irrelevant words, focus the attention of the model, and ultimately help to improve the performance of the whole model.

## 5. Conclusions

In this paper, we propose a novel model called Multi-modal Explicit Sparse Attention Networks (MESAN) for VQA. Considering many existing co-attention based VQA methods modeling dense interactions between each image region and each question word, which will force the models to calculate irrelevant information and have a negative impact on the performance of the models, MESAN reduces the interference from irrelevant information and focuses the attention of the model by using explicit selection based on top-k selection. Like other different sparse attention mechanisms used in NLP and CV fields, our models also achieve competitive results. A large number of ablation experiments and comparative experiments prove the effectiveness of our models and demonstrate that the models with sparse attention mechanisms can also achieve competitive results on VQA datasets. We also show the advantages of our model over other advanced VQA models through attention visualization. The limitation of this paper is that it is necessary to manually compare the different results of different *k* to select the best *k* value to achieve the best performance of the model. In the future studies, we will focus on exploring the method that can adaptively learn the optimal value of parameter *k* and strive to explore more effective sparse attention mechanisms for not only VQA, but also computer vision and natural language processing. We hope to propose better models to promote the application of VQA in intelligent transportation, smart city [45] and other artificial intelligence fields, and combine VQA with technologies based on the Internet of Things (IoT) Environment [46] to explore more application possibilities.

## Figures and Tables

**Table 1:** Ablation results of MESAN-SA on visual question answering (VQA) v2 test-dev set. The best results are bold.

*k*	Yes/No	Number	Other	All
3	86.72	53.83	60.48	70.54
4	86.96	52.97	60.58	70.58
5	86.97	52.79	60.70	60.62
6	86.91	**53.74**	60.65	70.68
7	86.85	53.35	60.63	70.61
8	**87.05**	53.21	**60.72**	**70.71**
9	87.02	52.81	60.62	70.61

**Table 2:** Ablation results of MESAN-SA&SGA on VQA v2 test-dev set. The best results are bold.

$k_{2}$	Yes/No	Number	Other	All
40	86.74	53.10	60.58	70.51
50	86.89	53.35	60.52	70.57
60	87.02	**53.43**	**60.63**	**70.68**
70	**87.04**	53.32	60.61	70.67
80	86.58	52.86	60.62	70.43

**Table 3:** Results of the state-of-the-art single models and our models on VQA v2. All the models use the same bottom-up attention visual features and are trained, validated, and tested on VQA v2. The best results on both splits are bold.

Model	Test-dev				Test-std
	Yes/No	Number	Other	All	All
Bottom-up [42]	81.82	44.21	56.05	65.32	65.67
MFH [16]	-	-	-	66.12	-
BAN + GloVe [5]	85.46	50.66	60.50	69.66	-
BAN + Glove + counter [5]	85.42	**54.04**	60.52	70.04	70.35
MCAN [23]	86.82	53.26	60.72	70.63	70.90
MEDAN(Adam) [24]	**87.10**	52.69	60.56	70.60	71.01
MESAN-SA(*k* = 8) (ours)	87.05	53.21	**60.72**	**70.71**	**71.08**
MESAN-SA&SGA($k_{1}=6,k_{2}=60$) (ours)	87.02	53.43	60.63	70.68	70.94

**Table 4:** Results of MEDAN and MEDAN-Sparse with different k∈ {4, 5, 6} on VQA v2 test-dev set. The best results are bold.

Model	Yes/No	Number	Other	All
MEDAN-Sparse(*k* = 4)	86.89	52.50	60.54	70.48
MEDAN-Sparse(*k* = 5)	86.95	**52.73**	**60.72**	**70.62**
MEDAN-Sparse(*k* = 6)	86.76	52.72	60.55	70.46
MEDAN(Adam)	**87.10**	52.69	60.56	70.60

## Abbreviations

The following abbreviations are used in this manuscript:

VQA	visual question answering
NLP	natural language processing
CV	computer vision
AI	artificial intelligence
DCN	dense co-attention network
BAN	bilinear attention network
MCAN	modular co-attention network
MEDAN	multi-modal encoder-decoder attention networks
MESAN	Multi-modal Explicit Sparse Attention Networks
DAN	Dual Attention Network
HieCoAtt	Hierarchical Co-attention Model
LSTM	long short-term memory
SA	self-attention
SGA	self&guided-attention
AMT	Amazon Mechanical Turk
MFH	generalized multi-modal factorized high-order pooling approach
MFB	Multi-modal Factorized Bilinear Pooling
IoT	Internet of Things
